# Prevalence of pregnancy experiences and contraceptive knowledge among single adults in a low socio-economic suburban community in Kuala Lumpur, Malaysia

**DOI:** 10.1186/1471-2458-14-S3-S1

**Published:** 2014-11-24

**Authors:** Li Ping Wong, Narges Atefi, Hazreen Abd Majid, Tin Tin Su

**Affiliations:** 1 Centre for Population Health (CePH), Department of Social and Preventive Medicine, Faculty of Medicine, University of Malaya, Kuala Lumpur, Malaysia; 2 Department of Nursing, Faculty of Medicine, University of Malaya, Kuala Lumpur, Malaysia

**Keywords:** Pregnancy, Single, Adult, Knowledge

## Abstract

**Background:**

This study aimed to investigate the prevalence of pregnancy experience and its association with contraceptive knowledge among single adults in a low socio-economic suburban community in Kuala Lumpur, Malaysia.

**Methods:**

A cross-sectional survey was conducted in 2012 among the Kerinchi suburban community. Of the total 3,716 individuals surveyed, young single adults between 18 and 35 years old were questioned with regard to their experience with unplanned pregnancy before marriage. Contraceptive knowledge was assessed by a series of questions on identification of method types and the affectivity of condoms for the prevention of sexually transmitted diseases.

**Results:**

A total of 226 female and 257 male participants completed the survey. In total, eight female (3.5%) participants reported experience with an unplanned pregnancy before marriage, and five male (1.9 %) participants had the experience of impregnating their partners. The participants had a mean total score of 3.15 (SD = 1.55) for contraceptive knowledge out of a possible maximum score of five. Female participants who had experienced an unplanned pregnancy had a significantly lower contraceptive knowledge score (2.10 ± 1.48) than who had never experienced pregnancy (3.30 ± 1.35), *p*<0.05. Likewise, male participants who had experienced impregnating their partners had a significantly lower contraceptive knowledge score (1.60 ± 1.50) than those who did not have such experience (3.02 ± 1.59), *p*<0.05.

**Conclusion:**

The results showed evidence of premarital unplanned pregnancy among this suburban community. The low level of contraceptive knowledge found in this study indicates the need for educational strategies designed to improve contraceptive knowledge.

## Background

Delay of sexual debut is an important strategy for reducing the risk of negative sexual adolescent health outcomes. It is reported that premarital sexual behaviour is not only increasingly accepted by young people in Asian countries but is also becoming more common in that a considerable proportion are engaging in premarital sex [1]. Unintended pregnancy during adolescence and the associated negative consequences of early pregnancy and early childbearing remain public health concerns. The consequences of premarital sexual involvement are damaging on many levels. On an emotional level they often include a profound sense of guilt, shame and regret. On a physical level they often include HIV, STIs, unwanted pregnancy and, on a social level, stigmatization [2,3].

A study from Malaysia's neighbouring country, Singapore, found that premarital sexual intercourse and permissiveness regarding premarital sex showed significant associations with living in low-cost housing [4]. Likewise, several studies from other developing countries have found that low or unstable income and poverty have been strongly linked to a higher likelihood of premarital sex among young people [5-8]. In a study conducted among in-school adolescents in Eastern Ethiopia, it was found that one in four, i.e., 686 (24.8%), reported pre-marital sexual debut. There were gender differences in terms of sexual intercourse, where males were more likely to have sexual initiation at an earlier age than females [9]. In Asia, a collaborative survey was conducted in urban and rural areas of Hanoi, Shanghai and Taipei among 16,554 unmarried participants aged between 15 and 24 years. The results of this study indicated that male respondents in each city had a more permissive attitude toward premarital sex than did females [10]. In Malaysia, it was found that approximately 12.6% of 1,139 students in an urban area between 15 and 20 years old reported having had a sexual experience: of these, 54.8% were male students [11]. Another cross sectional study conducted among 1,695 female university students aged 17 to 26 years found relatively poor reproduction and pregnancy knowledge among the participants [12]. It has also been found that exposure to the premarital sexual permissiveness of Western culture via mass media has greatly encouraged young people in Eastern and developing countries to engage in premarital sexual activity [13-15].

A number of studies reported a lack of contraceptive knowledge among adolescents who had undergone sexual initiation [16,17]. This is critical, as a lack of knowledge about how to prevent conception or failing to use contraception has been associated with the risk of unwanted pregnancy [18,19]. A study conducted among unmarried adolescents aged 10 to 19 in five locations in Tanzania showed that about 32% of adolescents reported being sexually active, but only 42% of them reported using a condom [20]. In the United States, every year there are 3.1 million unwanted pregnancies, 50% of which occur among women who have not used contraceptives in the month of conception [21].

A mixed method study conducted in Vietnam among a sample of 1,045 people aged 15 to 21 years found that participants' knowledge about contraceptive methods was low [22]. A survey carried out in 2011 by the Hong Kong Family Planning Association (FPAHK) among 1,126 unmarried youths aged 18 to 27 years found that approximately 41.5% of unmarried youths reported having engaged in premarital sex, whereas less than 10% engaged in sexual intercourse without protection: the majority of unmarried youths lacked contraceptive knowledge [1]. A study conducted among single individuals aged 15 to 30 years in Ghana showed that participants' knowledge about contraceptives types and their usage was low, indicating a lack of awareness about the various types of contraceptive methods [23].

It was also found that lack of knowledge on how to use contraceptive methods and the belief that modern contraception could affect fertility were associated with the non-use of contraception [24]. With regard to contraception methods, it has been reported that condoms and birth control pills were the most popular contraceptive methods used by adolescents [25,26]. Emergency contraception was reported to have considerable potential to reduce unintended pregnancy rates among teens; nevertheless, access to and knowledge of this form of contraception is limited among teens [27].

Young adults have one of the highest rates of unintended pregnancies and sexually STIs of any age group. Yet many sexually-active young adults rely on less effective contraceptive methods, [28] many use methods inconsistently or ineffectively [29] and many do not use any contraceptive method at all [28]. Given the high risk of this group, there is a clear need to identify ways in which to increase and improve contraceptive use.

This study aimed: 1) to investigate the prevalence of females' pregnancy experience and males' experience of impregnating their partners and 2) to discover the level of contraceptive knowledge among single adults in a low-income suburban community in Kuala Lumpur, Malaysia.

## Methods

A cross-sectional survey using anonymous, self-administered questionnaires was conducted in 2012 among the Kerinchi suburban community, where the majority of the population was low-income families. Firstly, all households were approached and invited to partake in the study. Households were selected to participate if an eligible participant was present at the time of visit. Single adults were eligible to participate if aged between 18 and 35 years and living in the household. If a household had more than one eligible participant, one eligible member was randomly selected to participate. Of the total 3,716 individuals from 833 households surveyed, young single adults between 18 and 35 years old were questioned with regard to their experience with unplanned pregnancy before marriage. The non-response data (age, gender, education) were obtained and compared with the response groups: findings revealed no significant difference.

Female participants were asked if they had pregnancy experience and male participants were asked if they had experience of impregnating their partners. Contraceptive knowledge was assessed by four questions which are as follows: the knowledge of participants relating to condoms, birth control pills, withdrawal and emergency contraception, and the effective use of condoms to prevent sexually transmitted infections. A correct response was given a score of one, and an incorrect or 'don't know' response was scored zero; the total possible maximum score was five.

The survey questionnaire was tested for face and content validity, and pilot-tested by a panel of experts. The questionnaire was in two languages: Bahasa Malaysia and English. All statistical analyses were performed with the Statistical Package for the Social Sciences Version 16.00 (SPSS; Chicago, IL USA). Non-responses and irrelevant answers were treated as missing values and, therefore, excluded from the analyses. A value of *p *≤ 0.05 was considered to be significant. T-tests and a one-way analysis of variance (ANOVA) were used for comparison of means.

### Ethical considerations

The study was approved by the Medical Ethics Committee of the University of Malaya Medical Centre, Kuala Lumpur, Malaysia (MEC Ref No. 890.161). Informed written consent was obtained from the participants, who were informed about the purpose and design of the study, and assured that participation was voluntary and confidential.

## Results

### Participants' characteristics

A total of 1,625 single adults were approached and 483 (response rate 29.7%) completed questionnaires were analysed. Details of the demographic characteristics of all the participants (n = 483) are summarised in Table 1. The ages of the participants ranged from 18 to 35 years, with a mean age of 23.25 years (SD ± 4.21). The majority of the participants were Malay (83%). More than half of the participants' (72.7%) average household incomes were below RM2000 monthly. Fewer than half of the participants (47.5%) had attained at least secondary education.

**Table 1:** Demographic characteristics of respondents (n = 483)

Characteristic	N (%)
**Age**	
18-25	363 (75)
26-35	120 (25)
**Gender**	
Male	257 (53.2)
Female	226 (46.8)
**Ethnicity**	
Malay	403 (83)
Chinese	5 (1.4)
Indian	75 (15.6)
**Religion**	
Muslim	409 (84)
Non-Muslim	74 (16)
Level of education *	
Primary school	192 (39.7)
Secondary school	281 (60.3)
**Average monthly household income**	
<2000	351 (72.7)
≥2000	132 (22.3)

*Not all subtotals add up to the total of 483 owing to missing values.

### Pregnancy experience

Of the total 483 completed responses, 226 were female and 257 were male. In total, eight female (3.5%) participants reported experience of unplanned pregnancy before marriage, and five males (1.9 %) had experience with impregnating their partners (Table 2). Three of those five had education only at the primary school level, four had fewer than RM2000 as their monthly income and all of them were Malay; more than half of the participants (n = 3, 60%) were between 26 and 35 years old. Among the eight female participants that had experienced pregnancy, two of them had only primary education, four had fewer than RM2000 as their monthly income, seven were Malay and the majority (n = 7, 87.5%) were between 18 and 26 years old. Four of the seven females with experience of pregnancy were under 20 years old.

**Table 2:** Distribution of socio-demographic characteristics and proportion of females who experiences pregnancy and males who experienced impregnating someone

	Frequency				Frequency (Female)	Experience of pregnancy			Frequency (Male)	Experience of impregnating someone		
			
Socio-demographic variables	(N = 483)	Yes	No	*p*	N (226)	Yes	No	*P*	N(257)	Yes	No	*P*
**Age **												
18-25	363	10 (2.8)	353 (97.2)	Ns	169	7 (4.1)	162 (95.9)	NS	194	3 (1.5)	191 (98.5)	NS
26-35	120	3 (2.3)	117 (97.7)		57	1 (1.8)	56 (98.2)		63	2 (3.2)	61 (96.8)	
**Ethnicity **												
Malay	403	12 (2.7)	398 (97.3)	Ns	189	7 (3.7)	182 (96.3)	NS	214	5 (2.3)	209 (97.7)	NS
Non-Malay	80	1 (1.3)	79 (98.7)		37	1 (2.7)	36 (97.3)		43	0	43 (100)	
**Religion **												
Muslim	409	12 (3)	397 (97)	NS	192	7 (3.7)	185 (96.3)	NS	217	5 (2.3)	212 (97.7)	NS
Non-Muslim	74	1 (1.4)	73 (98.6)		34	1 (2.9)	33 (97.1)		40	0	40 (100)	
Level of education *												
Primary school	192	5 (2.8)	187 (97.2)	Ns	97	2 (2.1)	95 (97.9)	NS	95	3 (3.2)	92 (96.8)	NS
Secondary school	281	8 (2.9)	273 (97.1)		129	6 (4.7)	123 (95.3)		152	2 (1.4)	150 (98.6)	
**Average monthly household income **												
<2000	351	8 (1.5)	343 (98.5)	Ns	164	4 (2.4)	160 (97.6)	NS	187	4 (2.1)	183 (97.9)	NS
≥2000	132	5 (3.8)	127 (96.2)		62	4 (6.5)	58 (93.5)		70	1 (1.5)	69 (98.5)	

*Not all subtotals add up to the total of 483 owing to missing values.

### Contraceptive knowledge

The results of the study indicated that condoms (87 %, n = 420) and birth control pills (82.4%, n = 397) were the most commonly known contraceptive methods for preventing pregnancy among all participants. Only 40% (n = 192) of all participants knew that withdrawal was a means of pregnancy prevention. Over a third (34.6 %, n = 167) of all participants knew that emergency contraception could prevent pregnancy.

There was no significant difference in the proportion of awareness about birth control pills for the prevention of pregnancy among female and male participants. A higher proportion of female participants (89.4%) than male participants (84.8%) were aware condoms could prevent pregnancy. Most of the female participants (88.1%) were aware that birth control pills could be used to prevent pregnancy compared with 77.4% of the male participants. Fewer than half of the female participants (39.8%) were aware that withdrawal could prevent pregnancy compared with 39.7% of the male participants. About 36.7% of female participants (n = 83) compared with 32.7% of the male participants (n = 84) knew about the emergency contraception pill (Table 3).

**Table 3:** Proportion of male participants' awareness of contraceptive methods compared with female participants

Contraceptive methods	Aware		Not aware		
	
	Female	Male	Female	Male	p
**Condom**	202 (89.4)	218 (84.4)	24 (10.6)	39 (15.2)	NS
**Birth control pills**	199 (88.1)	199 (77.4)	27 (11.9)	58 (22.6)	0.03
**Withdrawal**	90 (39.8)	102 (39.7)	136 (60.2)	155 (60.3)	NS
**Emergency contraception**	83 (36.7)	84 (32.7)	143 (63.6)	173 (67.3)	NS
**Condom can prevent sexually transmitted infection**	168 (74.3)	178 (69.3)	58 (24.7)	79 (30.7)	NS

When the knowledge was scored, overall the participants had a mean score of 3.15 (SD = 1.55) out of a possible maximum score of five. As shown in Table 4 significantly, female participants had a higher score on mean total contraceptive knowledge (3.28 ± 1.48) than male participants (3.00 ± 1.60), *p*<0.05). Among the female participants, there was no significant difference with regard to mean total knowledge score among the various demographic characteristics. There were also no significant differences with regard to mean total knowledge score between females with less than RM2000 as their monthly income and those who had more than RM2000 as their monthly income. Likewise, among the male participants, there were no significant differences in mean total knowledge score among the various demographic characteristics. There were also no significant differences in mean total knowledge score between males with less than RM2000 as their monthly income and those who had more than RM2000 as their monthly income.

**Table 4:** Socio-demographic characteristics: differences in mean total contraceptive knowledge score of all participants, female participants and male participants

Socio-demographic variables	Frequency of overall participants (%)	Mean ± SD		Frequency (Female)	Mean ± SD		Frequency (Male)	Mean ± SD	
		
	N (483)	(0-5)	*p*	N (226)	(0-5)	*p*	N(257)	(0-5)	*p*
**Age **									
18-25	363	3.10 (1.54)	NS	169	3.21 (1.50)	NS	194	2.99 (1.59)	NS
26-35	120	3.13 (1.59)		57	3.24 (1.29)		63	3.02 (1.50)	
**Gender**									
Male	257	3.02 (1.60)	0.03	−	−		−	−	
Female	226	3.28 (1.48)		−	−		−	−	
**Ethnicity **									
Malay	403	3.14 (1.56)	NS	189	3.29 (1.40)	NS	214	2.99 (1.49)	NS
Non-Malay	80	3.07 (1.48)		37	3.11 (1.52)		43	3.02 (1.52)	
**Religion **									
Muslim	409	3.12 (1.54)	NS	192	3.28 (1.56)	NS	217	2.97 (1.62)	NS
Non-Muslim	74	3.09 (1.49)		34	3.15 (1.45)		40	3.03 (1.50)	
Level of education *									
Primary school	192	3.08 (1.57)	NS	97	3.27 (1.51)	NS	95	2.89 (1.59)	NS
secondary school	281	3.17 (1.54)		129	3.26 (1.48)		152	3.09 (1.61)	
**Average monthly household income **									
<2000	351	3.12 (1.57)	NS	164	3.22 (1.57)	NS	187	3.02 (1.54)	NS
≥2000	132	3.01 (1.51)			62	3.10 (1.26)		70	2.92 (1.63)

*Not all subtotals add up to the total of 483 owing to missing values.

Female participants who had experienced unplanned pregnancy had a significantly lower contraceptive knowledge score (2.10 ± 1.48) than those who had never experienced pregnancy (3.30 ± 1.35). Likewise, male participants who had the experience of impregnating their partners had a significantly lower contraceptive knowledge score (1.60 ± 1.50) than those who had never done so (3.02 ± 1.59), *p*<0.05.

## Discussion

The results of this study showed that the prevalence of pregnancy among single adults was low in a low-economic suburban community in Malaysia compared with countries such as Hong Kong [1], Tanzania [20] and the United States [21]. The study has shown evidence that unmarried young people have experienced sexual intercourse, even though premarital sexual intercourse is prohibited among Muslims. Despite its low prevalence of extra-marital pregnancy, the incidence of extra-marital pregnancy in this Muslim majority community warrants considerable attention. Most of the participants who had experienced premarital pregnancy were from households with fewer than RM2000 as their monthly income and of a lower educational level. Of particular concern is that many of those with pregnancy experience were under the age of 20 years old. This may imply that premarital sex and premarital pregnancy experience are common among the less socio-economically advantaged, as found in other studies [3-7]. These results may suggest that there should be interventions to raise conservative attitudes toward premarital sex and preventive interventions to target the lower income group of the low socio-economic community.

Consistent with other studies [25,26], the condom was the most well-known contraceptive method by all participants. Emergency contraception could play a critical role in reducing unintended pregnancies [26,30,31]; however, many studies found that the awareness and use of emergency contraception was often low among women in India, Latin America and African countries [30-32]. Likewise, our results showed that emergency contraception pills were not known by most of the participants as a method for preventing pregnancy. Therefore, it is essential to increase knowledge about emergency contraception methods among the community.

With regard to withdrawal as a pregnancy prevention method, like another study conducted in Asia, [33] the results of this study show that most participants were not aware of this method, particularly females. This is an area that needs to be addressed in the development of sex education [33].

On the whole, this study found inadequacy in basic knowledge about contraceptive types among the youth in the community studied. This is of particular concern as poor knowledge of young people about contraceptives carries risks of unplanned premarital pregnancy, impregnation or STIs [27,30]. Young people should be educated about contraceptive methods and pregnancy prevention, especially the males in the community where level of knowledge about contraceptive was found to be low. Females were, on the whole, more knowledgeable about contraceptive methods in this study, as in [34]. Since males are perceived to have more decision-making power than their female counterparts, it is important to enhance the level of contraceptive knowledge and encourage male involvement in reproductive health programmes, as this may contribute to the reduction of unwanted pregnancies [35].

Our findings revealed that female participants who experienced unplanned pregnancy had a significantly lower contraceptive knowledge score than those who had never experienced pregnancy. Likewise, male participants who had experienced impregnating their partners had a significantly lower contraceptive knowledge score than those who had never done so. Other studies also reported that contraceptive knowledge among adolescents with experience with premarital sex was deficient [16,17]. This suggests that low contraceptive knowledge may be associated with pregnancy experience. The low level of knowledge about contraception among young people with experience of pregnancy or impregnation showed that educational and behavioural interventions are urgently needed to prevent STIs. The study found a higher level of contraceptive knowledge among females than males, implying that intervention should particularly target males. This study has yielded considerable insight into the contraceptive knowledge among single adults in a low socio-economic suburban community. These findings also have important implications for the development of effective sexual and reproductive educational programmes among this population.

This study has several limitations. The first limitation relates to the relatively small size of the study sample. For this reason, these findings cannot be generalised to the broader community, thus, further research with bigger sample sizes would increase the confidence in the robustness of the results found in this study. The next two limitations are related to the sensitive topic of the research where participants were asked about their sexual behaviour. Bias introduced by under-reporting is possible as premarital sex is a sensitive issue and discussion of this issue may be considered socially unacceptable in Malaysian cultural settings. A poor response rate associated with the sensitivity of the questions may lead to sample bias.

## Conclusion

Results have shown evidence of premarital unplanned pregnancy among the suburban low socio-economic community investigated. The results of this study indicated that contraceptive knowledge among young, single adults was moderate. In addition, the lower level of contraceptive knowledge among females who had experienced unplanned pregnancy than those who had never experienced pregnancy in this study indicates a need for educational strategies designed to improve contraceptive knowledge among this target group. In this study, emergency contraception pills were not known by most of the participants as a method for preventing pregnancy. More educational programmes are required to increase knowledge about emergency contraception methods within the community. The results of this study also showed that many who had a premarital pregnancy were from households within the lowest income group of this low socio-economic sub-urban community.

Further detailed studies with larger sample sizes are required to assess the risk of sexual behaviour among young singles and also the factors related to family and parents, such as how parental attitudes influence the practice of premarital sex. In addition, due to the sensitive nature of the questions, further qualitative study may be required to find out more in-depth information about sexual experience and knowledge regarding contraceptive methods among young, single adults in suburban communities.

## List of abbreviations

HIV, Human Immunodeficiency Virus; STIs, Sexually Transmitted Infections; FPAHK, Hong Kong Family Planning Association; ANOVA, One-way Analysis of Variance.

## Competing interests

The authors declare that they have no competing interests.

## Authors' contributions

LPW coordinated the study, gave substantial input into data analysis and critically reviewed the manuscript. TTS and HAM involved in data collection. NA performed the statistical data analysis. All authors contributed to and have approved the final manuscript.
